# New Polymer Biocomposites Based on Biopoly(Ethylene Terephthalate) and Waste Mollusc Shells

**DOI:** 10.3390/ma17194752

**Published:** 2024-09-27

**Authors:** Stanisław Kuciel, Karina Rusin-Żurek, Maria Kurańska

**Affiliations:** 1 Faculty of Materials Engineering and Physics, Cracow University of Technology, al. Jana Pawła II, 31-864 Kraków, Poland; karina.zurek@doktorant.pk.edu.pl; 2 Faculty of Chemical Engineering and Technology, Cracow University of Technology, Warszawska 24, 31-155 Kraków, Poland

**Keywords:** bPET, circular economy, biocomposites, mollusc shells

## Abstract

Currently, scientific studies have are focusing on environmentally friendly solutions, such as the effective use of waste in new green polymeric materials according to circular economy. Waste valorization is the main driving force for upcoming academic research. In this study, the impact of mussel particle size on reinforced biopoly(terphtalate ethylene) (bPET) is investigated. The waste filler was modified using NaOH. The filler content was 10 wt% and the same for all samples. The strength properties of the materials were determined in static tensile, bending and impact tests. The wetting angle was also analyzed for the obtained biocomposites. A low-cycle dynamic test was carried out to determine changes in dissipation energy and to observe the development of relaxation processes. This present study proves that preparation of new biocomposites based on waste mussels is an effective option in waste management.

## 1. Introduction

These days, the circular economy plays a key role in the development of every industry branch. It is a model of production and consumption that involves sharing, borrowing, reusing, repairing, refurbishing, and recycling existing materials and products for as long as possible. The aim is to minimize waste. When a product reaches the end of its useful life, recycling should preserve the leftover raw materials in the economy. This idea is in line with new circular economy-related trends to find alternative components for polymer composite synthesis based on waste. Composites are new generation materials that have been designed to meet the demands of rapid technological development in industry. The matrix of composites can be either polymers of petrochemical origin or polymers from renewable raw materials.

In 2022, more than 400 million tons of plastic were produced worldwide, of which only 2.3 Mt were bioplastics [1,2]. Bioplastics are plastics produced from natural resources through chemical processes or through biosynthesis by living organisms. Biopolymer materials have attracted attention, owing to their availability, sustainability, environmental friendliness, and biodegradability.

Polymer materials based on petrochemical resources are not environmentally friendly. That is why governments and researchers have begun to give importance to the production of biopolymers with organic fillers that can be alternatives to traditional materials. Various studies on incorporating biofillers into the polymer matrix have been carried out so far [2,3,4,5,6,7,8,9]. Biopoly(ethylene terephthalate) (bioPET) is a type of thermoplastic polymer that is made from biological raw materials. Currently, the content of renewable substances in bioPET is about 30%, which is related to the possibility of obtaining ethylene glycol from renewable raw materials. However, there is a future possibility of obtaining bioPET fully from renewable raw materials thanks to the existence of a biological path of terephthalic acid synthesis [10].

The world production of mussels in the last twenty years has increased rapidly. The global production of bivalve molluscs, taking into account inland, marine, and coastal aquaculture, is equivalent to 17.7 million tons [11]. Mussels are food proteins extensively consumed by humans around the world, and the majority of their shells are disposed of in landfills. Shell wastes constitute a substantial amount of the by-products in shellfish aquaculture. For eco-friendly and economical recycling, it is desirable to convert these residues into high value-added fillers. Bivalve molluscs make up almost 10% of the world’s total fishery production, while mussels form 14% of mollusc production via aquaculture. Shells make up 75 to 90% of the entire amount of mussel waste produced [12]. In line with the idea of a closed-loop economy, waste shells could be used as fillers for polymer materials [13,14,15,16]. This approach reduces the share of the polymer in the finished product. 

Shell waste consists predominantly of CaCO_3_ (~95%) [17]. Besides CaCO_3_, there also exists around 5% of organic materials, including glycoproteins, polysaccharides, chitin, and other proteins [18,19,20]. 

Moustafa et al. proposed the use of ground waste seashells as a biofiller to reinforce the acrylonitrile–butadiene–styrene copolymer (ABS). The waste seashell filler was added to ABS to enhance the mechanical properties, thermal stability, and flame-retardant properties [18]. Chong et al. prepared plastic materials from polyethylene and surface-coated oyster shells. The composites exhibited better mechanical properties and fire-retardant behavior than neat polyethylene [21]. Hamester modified polypropylene with calcium carbonate obtained from oyster and mussel shells and concluded that it is technically possible to replace commercial CaCO_3_ with that obtained from the shells of shellfish in polypropylene composites [20]. Karthick evaluated the hardness and tribological properties of a poly(ethyl methacrylate) (PMMA) based denture composite reinforced with seashell nanopowder. PMMA biocomposites containing 2%, 4%, 6%, 8%, 12%, 16%, and 20% by weight of seashell nanopowder and an unfilled composite as a control specimen were obtained. It was concluded that PMMA biocomposites could be successfully reinforced by seashell nanopowder, with the best properties obtained with 12% and 8% seashell nanopowder contents [20]. Gigante et al. obtained composites based on a compostable matrix made of polylactic acid (PLA) and poly(butylene adipate-co-terephthalate) (PBAT). The effect of an increasing content of mussel shell powder was investigated in the range from 5 to 20 wt% [12].

To the best of our knowledge, no studies on bioPET modification with waste fillers have been described in the literature so far. However, the issue is important from the point of view of implementing the principles of the circular economy in plastics technology. The aim of this work was to analyze the possibility of using shells as a substitution of bioPET. For this purpose, fillers with different particle sizes were prepared and their influence on basic functional properties was assessed. This research is related to the development of modern polymer composites based on bio-raw materials modified with waste fillers in accordance with the concept of a circular economy.

## 2. Materials and Methods

### 2.1. Materials

The materials used in this work were the following:

- Polymer matrix: bioPET NP002 purchased from Nature Plast Mondeville (France).
- Waste filler: mollusc shells were initially soaked for 2 weeks in a solution of water and dish soap and then rinsed thoroughly. Later, they were treated with a 10% NaOH solution for 45 min and rinsed again. In the next stage, they were boiled for 30 min. The dried shells were then ground in a disc mill (FRITSCH, PULVERSIETTE 13). Subsequently, the particle size was analyzed using a vibration shaker (FRITSCH, analysette 3) and 4 fractions were separated: (1) particles larger than 3 mm—these particles were rejected; (2) Particles between 1.60 and 3 mm—large particles (MSb); (3) Particles between 1.60 and 1 mm—medium particles (MSm); (4) Particles between 1 mm and 200 μm—small particles (MSs). The filler content in all samples was 10% mass.
- Compatibilizers: Scona TPPL 5112PA; Scona TPKD 8304 PCC; and Scona TPKD 8304 PCC purchased from BYK-Chemie Gmb (Wesel, Germany).

### 2.2. Preparation of Composites

Composite pellets were produced using a compounding line with a Steer Omega 20 H (Bangalore, India) co-rotating twin screw laboratory extruder. The process was carried out at 270 °C and the screw speed was set to 90 rpm. A compatibilizer was added at an amount of 3%. Tests were carried out with the three compounds mentioned above. Based on preliminary tests (Table 1), Scona TPPL 5112PA was selected for further research.

Samples for mechanical tests were obtained by injection molding (KM 40-125 Winner Krauss Maffei, Krauss Maffei, Munich, Germany) in accordance with PN-EN ISO 3167. The process was carried out at a temperature profile of 200–280–285–290–295 °C, and an injection pressure of 900 bar.

### 2.3. Testing Methods

A particle size analysis was performed using an Anton-Paar PSA 1190LD laser particle size analyzer. The test was conducted using the wet method with distilled water as a dispersing agent. Four measurements were carried out for each material, and then the average of the results was calculated using Kalliope Professional software. 

An optical microscope Keyence VHX 5000 (Osaka, Japan) dedicated to digital image analyses was used to analyze the organic waste particles at 50×, 100×, and 200× magnifications. The morphology of composites was investigated by means of a 20 kV scanning electron microscope (SEM (JEOLJSM5510LV, Tokyo, Japan)). Prior to SEM, samples were coated with a thin layer of gold, under a vacuum, using an auto vacuum coater (Cressington, Watford, UK) to avoid electrical charge accumulation.

Static tensile tests and low-cycle dynamic tests were carried out on a Shimadzu AGS-X 10 kN testing machine (Kyoto, Japan). The tensile test was performed in accordance with the PN-EN ISO 527–1 standard at a cross-head speed of 10 mm/min. In the dynamic test, a cyclically loaded deformation from a minimum value of 400 N to a maximum of 1200 N at a speed of 5 mm/min was applied. Flexural properties were determined on a universal testing machine MTS Criterion 43 (Massachusetts, MN, USA) with MTS TestSuites 1.0 software in accordance with PN-EN ISO 178 at a cross-head speed of 10 mm/min. Charpy impact strength was measured using a Zwick/Roell testing machine (Ulm, Germany). An impact energy of 5 J was used for unnotched samples in accordance with PN-EN ISO 179–1.

The contact angle and surface free energy were measured using a goniometer (See System, Advex Instruments, Brno-Komín, Czech Republic) on the surfaces of the composites. Water and diiodomethane were used in the measurements.

## 3. Results and Discussion

### 3.1. Properties of Modified MS

**Figure 1:**
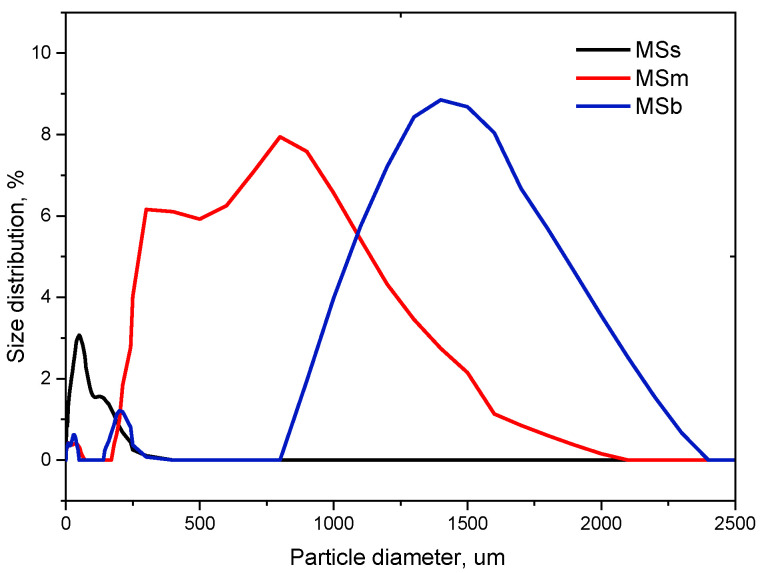
Particle size distribution curves of MS.

Three fractions of ground MS were used to modify bioPET: small (MSs), medium (MSm), and big (MSb). Figure 1 shows MS particle size distribution curves. Table 2 presents the results of the particle size analysis.

**Figure 2:**
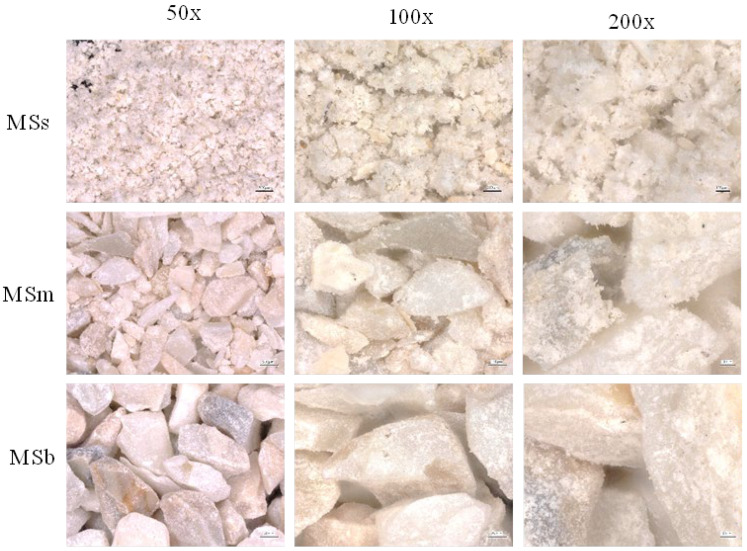
Optical microscope images of organic waste fillers at different magnifications: 50×, 100×, and 200×.

MSs are characterized by an average particle size of 45 µm. For MSm and Msb, the average particle sizes are 651 and 1136 µm, respectively. The broadest peak in the particle size (particle diameter) distribution was observed for medium-sized particles, which is a consequence of a wide range of the adopted definition. A confirmation of the presence of two maxima of the peak can be found in optical images visualizing medium-sized particles. In the images, one will notice the occurrence of two kinds of particles: those with sizes up to 500 μm and those around 700–800 μm. Optical microscope photographs are presented in Figure 2.

### 3.2. Mechanical Properties of the Biocomposites

**Figure 3:**
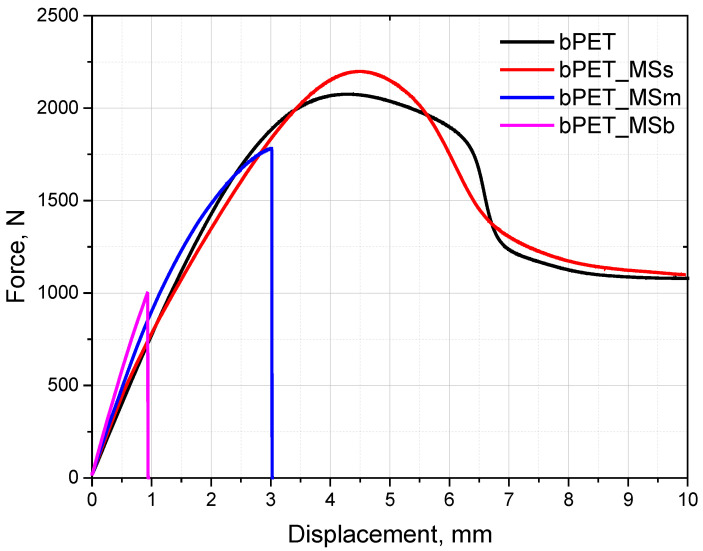
Tensile strength curves of bioPET composites.

**Figure 4:**
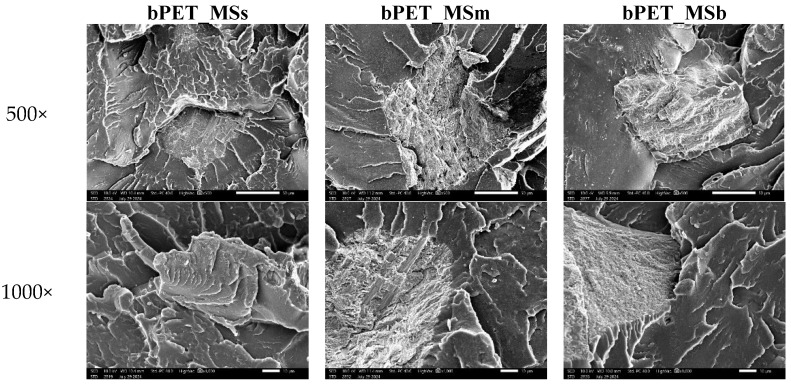
SEM micrographs of compatibilized bioPET-based composites after tensile tests.

**Figure 5:**
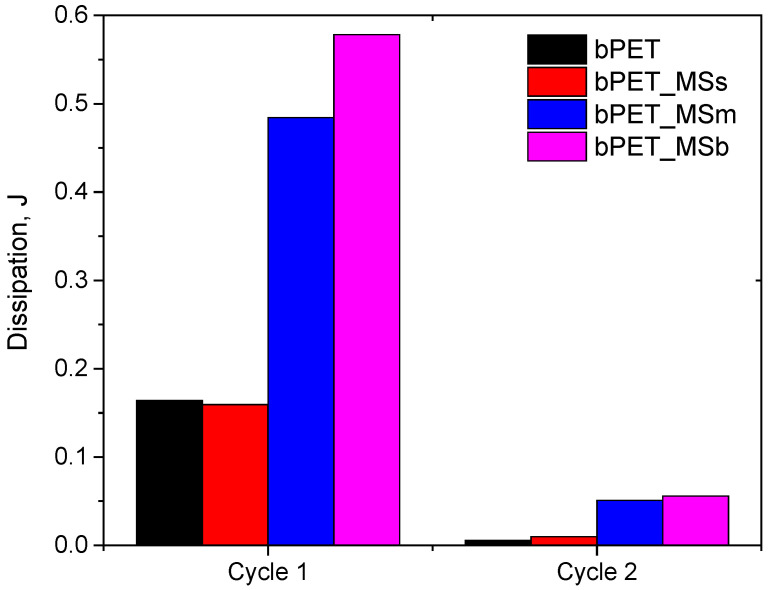
Comparison of mechanical energy dissipation values for cycle 1 and 2.

The results concerning mechanical properties of the static tensile and three-point bending tests are presented in Figure 3, Figure 4 and Figure 5 as well as Table 2 and Table 3.

It was found that an addition of MSs increases the tensile strength by approximately 5% and the elastic modulus by 17%. Increasing the amount of MS leads to stiffening of the biocomposites, confirmed by an increase in the elastic modulus. Since the elastic modulus of inorganic particles is usually much higher than that of a polymer, the elastic modulus of a composite (made of such particles and polymer) is expected to be higher than that of the polymer matrix. The biocomposites with MS confirm this expectation. The plain strain indentation modulus of single calcite crystals from mollusc shells can reach values of about 75 GPa [12]. The highest impact strength values were also observed for the composites with the smallest particles. Figure 3 shows that the larger the shell particle, the lower the maximum force and the smaller the deformation at break. The results indicate lower adhesion of large particles and their detachment from the polymer matrix under a stretching stress (Figure 4). In the case of bending strength, where the direction of force is perpendicular to the particles arranged along the injection matrix, their size causes the formation of internal stresses at the particle–polymer boundary, which results in the smallest reduction in bending strength and an increase in the bending modulus by 30% (Table 4). In the case of bending strength, where the direction of force is perpendicular to the particles arranged along the injection axis, their size causes the formation of internal stresses at the particle–polymer boundary. Such stresses result in the smallest observed reduction in the bending strength and an increase in the bending modulus by 30% (Table 4).

**Figure 6:**
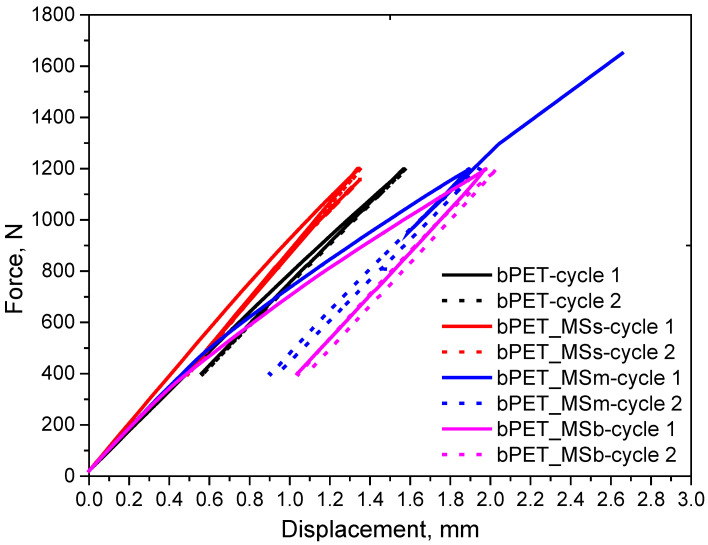
Mechanical hysteresis loops for cycle 1 (solid line) and cycle 2 (dashed line).

An analysis of mechanical energy dissipation was carried out for the first two loops with dynamic forcing in the force range of 400–1200 N. It was found that an addition of small particles reduced the ability to dissipate it (Figure 5). This is related to a reduction in internal friction processes and a lower ability to generate heat, which leads to an increase in permanent fatigue strength. This effect is confirmed by the hysteresis curves shown in Figure 6, where we can observe the lowest displacement capacity at maximum force for the composite with small particles and the highest one for the composite with large particles. Large particles of waste shells are not well embedded in the polymer matrix, which destroys adhesive connections. This leads to an increase in dynamic creep processes already present in the first loops of mechanical hysteresis.

Wettability is a feature of materials that determines their interaction with liquids, mainly water, and their basic properties, e.g., adhesion. A measure of surface wettability is wetting angle θ (also known as contact angle or critical angle), formed between the wetted surface of a solid and a tangent to the surface of the wetting liquid (curvature of the meniscus of the wetting liquid), derived from the point of contact of the liquid with the surface of the solid. The contact angle and total surface free energy found in our tests are presented in Table 5.

The addition of waste particles decreased the contact angle and increased the total surface energy. The mechanical tests confirmed the resultant lower adhesion of the particles to the matrix.

## 4. Conclusions

Our results show that a proper choice of compatibilizer and waste shell particle size matters for improving strength and, most importantly, stiffness. Small shell particles forming crystallization nuclei exhibit the most advantageous mechanical properties. Smaller particles are characterized by a larger surface area and, thanks to the compatibilizer, the interfacial adhesion forces increase strength and stiffness. Larger particles have a smaller surface area and the forces between that surface and the matrix are greater given a large deformation capacity of the matrix. Therefore, the total adhesion from the smaller specific surface area is lower. It is therefore possible to utilize shellfish waste from restaurants and replace synthetic fibers, e.g., glass or polymer, while making polymeric composites boasting better strength properties. This study confirms the suitability of waste shells as a filler in composites with bio-based PET. It allows for the production of a highly efficient composite with a carbon footprint reduced by the use of not only a bio-based matrix but also a waste filler. Further studies should assess the dynamic creep ability of such composites and characterize their fatigue strength.

## Figures and Tables

**Table 1:** Tensile strength properties of samples containing compatibilizers.

Compatibilizer	Tensile Strength [MPa]	Young’s Modulus [MPa]	Max. Displacement [mm]
Scona TPPL 5112 PA	66.7 ± 3.36	4327 ± 297	3.96 ± 0.32
Scona TPKD 8304 PCC	64.7 ± 4.74	4236 ± 69	4.04 ± 0.63
Scona TPKD 8304 PCC	52.8 ± 0.19	4336 ± 88	3.23 ± 0.05

**Table 2:** Particle size analysis results of mollusc shells.

Particles	D10 [µm]	D50 [µm]	D90 [µm]	Average Value [µm]
MSs	2.64 ± 0.12	28.05 ± 1.51	107.44 ± 7.133	44.75 ± 2.13
MSm	38.93 ± 8.23	546.58 ± 33.33	1149.09 ± 34.14	651.26 ± 26.02
MSb	13.21 ± 4.66	1219.41 ± 36.66	1819.77 ± 45.48	1135.61 ± 58.74

**Table 3:** Tensile strength properties of bio-based PET composites with additions of MS.

Sample	Tensile Strength [MPa]	Young’s Modulus [MPa]	Max. Displacement [mm]	Impact Strength [kJ/m^2^]
bPET	52.1 ± 0.37	2871 ± 231	27.7 ± 3.5	does not break
bPET_MSs	54.9 ± 0.60	3367 ± 33	8.7 ± 1.6	14.53 ± 1.06
bPET_MSm	39.9 ± 4.57	3748 ± 248	2.3 ± 0.6	9.09 ± 1.14
bPET_MSb	24.9 ± 1.24	3377 ± 168	0.9 ± 0.1	4.9 ± 0.92

**Table 4:** Bending strength properties of bio-based PET composites with additions of MS.

Sample	Flexural Strength [MPa]	Flexural Modulus [MPa]	Displacement [mm]
bPET	76.3 ± 2.54	1949 ± 64	>10
bPET_MSs	63.6 ± 0.52	2596 ± 253	>10
bPET_MSm	68.1 ± 1.13	1853 ± 13	>10
bPET_MSb	72.6 ± 2.11	2002 ± 72	>10

**Table 5:** Contact angle and total surface free energy by the Owens–Wendt method.

Sample	Contact Angle θ in Degrees		
	Water	Diiodomethane	Total Surface Free Energy [mJ/m^2^]
bPET	88.0 ± 4.5	37.0 ± 0.5	42.27 ± 2.1
bPET_MSs	64.4 ± 1.6	31.4 ± 2.6	52.50 ± 1.9
bPET_MSm	77.4 ± 1.4	32.5 ± 3.3	46.83 ± 2.8
bPET_MSb	67.5 ± 1.7	35.1 ± 2.5	49.20 ± 1.8

## Data Availability

No new data were created or analyzed in this study. Data sharing is not applicable to this article.
